# Determination of Two Antiepileptic Drugs in Urine by Homogenous Liquid-Liquid Extraction Performed in A Narrow Tube Combined with Dispersive Liquid-liquid Microextraction Followed by Gas Chromatography-flame Ionization Detection

**DOI:** 10.22037/ijpr.2019.1100635

**Published:** 2019

**Authors:** Behruz Feriduni, Mir Ali Farajzadeh, Abolghasem Jouyban

**Affiliations:** a *Pharmaceutical Analysis Research Center, Faculty of Pharmacy, Tabriz University of Medical Sciences, Tabriz, Iran. *; b *Department of Analytical Chemistry, Faculty of Chemistry, University of Tabriz, Tabriz, Iran.*; c *Department of Engineering Chemistry, Faculty of Engineering, Near East University, 99138 Nicosia, North Cyprus, Mersin 10, Turkey.*; d *Kimia Idea Pardaz Azarbayjan (KIPA) Science Based Company, Tabriz University of Medical Sciences, Tabriz, Iran.*

**Keywords:** Gas chromatography, Antiepileptic drugs, Urine, Homogenous liquid-liquid microextraction, Dispersive liquid-liquid microextraction

## Abstract

A simple and efficient homogenous liquid-liquid extraction method performed in a narrow tube combined with dispersive liquid-liquid microextraction method has been presented for the simultaneous determination of two antiepileptic drugs in urine followed by gas chromatography with flame ionization detection. In this method, a mixture of acetonitrile and urine sample (homogenous solution) is loaded into a column partially filled with solid sodium chloride. By passing the homogenous solution through the salt layer, acetonitrile is separated from the aqueous solution as the fine droplets and collected on top of the column as a separated phase. The obtained organic phase is removed and mixed with an extraction solvent, and then the resulting mixture is rapidly injected into an alkaline solution. Various experimental parameters affecting performance of the proposed method such as type and volume of extraction solvent, pH, and flow rate in homogenous liquid-liquid extraction step, and type and volume of extraction solvent and ionic strength in dispersive liquid-liquid microextraction step were investigated. The relative standard deviation of the proposed method was <8% (n = 6, C = 1 µg L^-1^ of each analyte). The limits of detection for phenobarbital and carbamazepine were 0.017 and 0.010 µg L^-1^ and the limits of quantification were 0.056 and 0.033 µg mL-^1^, respectively.

## Introduction

Therapeutic drug monitoring (TDM) of antiepileptic drugs (AEDs) is an accepted practice because (a) physiological markers for clinical effects of AED efficacy or toxicity are not immediately apparent, (b) clinical response corresponds better to concentrations of drugs than doses, and (c) seizures occur at irregular intervals, making treatment prophylactic and dosing changes a challenge. In addition, treatment is usually lifelong, with toxicity avoidance of utmost importance (1, 2). Suggested target ranges of AEDs were established only after the development of accurate and sensitive analytical methods. Several analytical procedures have been described for the measurement of AEDs including immune assays, high-performance liquid chromatography, gas chromatography (GC), and capillary electrophoresis (3-14). Biological sample matrices such as urine and plasma are complicated and often contain the compounds which can interfere with the compounds of interest, so that direct analysis may not be possible. Moreover, pharmaceuticals are generally found in these matrices at trace concentration levels. Therefore, it is necessary to perform an initial sample-preparation step, including purification and concentration of the analytes. Also, this step converts the sample into a suitable phase for analysis. Sample preparation may be achieved by a wide range of techniques but all of them show the above-mentioned goals, in addition to provide a robust and repeatable reproducible method which is independent of variations in the sample matrix (15). Even though traditional sample preparation methods such as solid phase extraction and liquid-liquid extraction are still in use, these methods are labor and tedious and they require large volume of biological samples (16, 17). Thus, trends in recent years are focused towards small initial sample size, small volume or no organic solvent, greater specificity or greater selectivity in extraction and increased potential for automation. Therefore, sample preparation methods with the mentioned properties such as solid phase microextraction (SPME) and liquid phase microextraction (LPME) were considered (18-20). SPME by combining extraction and preconcentration in a single step, is an effective and solvent–free technique. However, SPME fibers are relatively expensive, generally fragile, and have a limited lifetime, especially for some direct immersion extraction from complex sample matrices. Dispersive liquid-liquid microextraction (DLLME) is a version of LPME which was applied for extraction and preconcentration of drugs from biological samples (21-23). This method is based on a ternary solvent system, in which a solution of an extraction solvent in a disperser solvent is rapidly injected into an aqueous sample by a syringe. The disperser solvent must be fully miscible with both the aqueous sample and the extraction solvent. The extraction solvent should be insoluble in water and must have a density different from that of aqueous sample. Some advantages of the DLLME are simplicity of operation, low cost, rapidity, and high enrichment factors. It has attracted much attention by many research groups.

In this paper, a simple and efficient method was developed for extraction, preconcentration, and determination of two antiepileptic drugs including phenobarbital (PB) and carbamazepine (CBZ) in urine. In this method, a mixture of acetonitrile (ACN) and urine sample (homogenous solution) was loaded into a column which a portion of it was filled with sodium chloride. By dissolution of sodium chloride into the sample ACN was separated via salt-induced homogenous liquid-liquid extraction (HLLE). The obtained organic phase was removed and used in the following DLLME procedure. 

## Experimental

*Reagents and solutions*

The selected antiepileptic drugs including PB and CBZ were gifts from AminPharmaceutical Company (Tehran, Iran) and Arastoo Company (Tehran, Iran), respectively. Solvent such as ACN, dimethyl formamide (DMF), carbon tetrachloride, acetone, chloroform, methanol, 1,1,2–trichloroethane (1,1,2–TCE), and 1,1,2,2-tetrachloroethane (1,1,2,2-TCE) were obtained from Merck (Darmstadt, Germany). Sodium chloride, sodium hydroxide, and hydrochloric acid were purchased from Merck. Isopropyl alcohol was from Caledon (Canada). Deionized water was obtained from Ghazi Company (Tabriz, Iran). A stock solution of the studied drugs was prepared by dissolving appropriate amounts of the analytes in methanol at a concentration of 1000 mg L^–1^ of each drug. Working standard solutions were prepared daily by diluting the stock solution. A mixture of standard solution of the analytes (1000 mg L^–1 ^of each drug) in chloroform (extraction solvent) was prepared and directly injected into the separation system each day (three times) in order to evaluate the instrumental system quality and to calculate enrichment factors (EFs) and extraction recoveries (ERs) of the analytes.

*Real samples*

Urine samples were obtained from volunteers working in our laboratory. It is noted that none of the volunteer did not receive the studied drugs. One of the urine samples was used as a blank urine in the optimization step. The matrix effect was reduced through dilution of the samples with deionized water at a ratio of 1:1 and subsequently the samples were subjected to HLLE-DLLME procedure and GC measurement.

*Instrumentation*

GC analysis of the analytes was carried out using an Agilent 7890A gas chromatograph (Agilent Technologies, CA, USA) equipped with a split/splitless inlet operated at 300 °C in a splitless/split mode (sampling time 1 min and split ratio of 1:2) and a flame ionization detector (FID). Nitrogen (99.999%, Gulf Cryo, United Arabic Emirates) was used as the carrier gas (at a constant flow rate of 1 mL min^−1^) and make up gas (25 mL min^−1^). Chromatographic separation was achieved on an HP**–**5 capillary column (30 m × 0.32 mm i.d., with a 0.25 µm stationary film thickness) (Hewlett-Packard, Santa Clara, USA). The oven temperature was programmed from 70 °C (held for 2 min) to 300 °C at a rate of 10 °C min^−1^ and held at 300 °C for 3 min. Chem Station software was used for data acquisition and processing. A 1-μL microsyringe (zero dead volume, Hamilton, Switzerland) was used for the injection of samples into GC. The FID temperature was maintained at 300 °C. Hydrogen gas was generated with a hydrogen generator (GLAIND-2200, Dani, Italy) for FID at a flow rate of 40 mL min^−1^. Air flow rate was 400 mL min^-1^. A vortex from Labtron Company (Tehran, Iran) was used in sample preparation. A Metrohm pH meter model 744 (Herisau, Switzerland) was used for pH measurements. Hettich centrifuge (Tuttlingen, Germany) was used for accelerating phase separation.

*Procedure*

Initially, the end of tube (12 ×1 cm i.d.) was connected to a stopcock and 2 g sodium chloride was filled into the tube. A 5 mL of diluted blank urine spiked with the selected analytes (1 mg L^-1^ of each analyte) was mixed with 2 mL ACN and a homogenous solution was obtained. In the following, the homogenous solution was poured into the tube. The solution was passed through the salt at a flow rate of 1 mL min^-1^. By passing the homogenous solution (urine + ACN) through the tube, the fine droplets of ACN were formed in the interface of solid (NaCl) and solution due to dissolution of the salt into the solution (salting–out effect). The produced droplets moved through the remained solution to top of the narrow tube and floated on the surface of solution as a separated layer due to lower density of ACN (d = 0.786 g mL^–1^) with respect to water. During this step, the analytes were extracted into the ACN. After passing all aqueous solution, the stopcock was closed. Volume of the separated phase (ACN) on the top of the remained solid NaCl was 1.00 ± 0.05 mL. Then, the collected organic phase was removed and mixed with 40 µL chloroform. Then, the obtained organic phase was rapidly injected into 5 mL deionized water (pH adjusted to 10 by sodium hydroxide) containing 10% w/v, sodium chloride by 5 mL syringe. A cloudy solution, resulted from dispersion of the tiny droplets of chloroform into the aqueous solution due to dissolving ACN into water, was formed and the drugs were extracted and concentrated into chloroform. The mixture was then centrifuged for 5 min at 5000 rpm, which led to settle down the dispersed droplets of the extractant at the bottom of the tube. After centrifuging 10 ± 0.5 µL of the settled organic phase was obtained. Finally, an aliquot (1 µL) of the organic phase was removed and injected into the separation system for analysis 

*Calculation of EF and ER*

The EF and ER were used as the parameters to evaluate the method efficiency. They were calculated by Equations 1 and 2, respectively.

$\mathrm{EF}=\frac{C_{\mathrm{sed}}}{C_{0}}$

(1)

where C_sed_ and C_0_ are the concentrations of the analytes in the final organic phase obtained in DLLME step and the initial concentration of the analytes in the diluted urine sample, respectively.

$\mathrm{ER\%}=\frac{n_{\mathrm{sed}}}{n_{0}}\times 100$ = $\frac{C_{\mathrm{sed}\times V_{\mathrm{sed}}}}{C_{0\times V_{\mathrm{aq}}}}$ × 100 = EF × $\frac{V_{\mathrm{sed}}}{V_{\mathrm{aq}}}$× 100                (2)

where V_sed_ and V_0_ are the volumes of the final sedimented organic phase and urine sample, respectively. The n_sed_ and n_0_ are the extracted and initial amounts of the analytes, 

respectively. 

## Results and Discussion

In this work, a combination of HLLE performed in narrow tube and DLLME was developed for the determination of low levels of AEDs in urine samples. In order to find the best experimental conditions for the proposed method, a step-by-step optimization was used. Some variables, affecting the performance of the experimental procedure, such as type and volume of extraction solvent, pH, and flow rate in HLLE step and type and volume of extraction solvent, ionic strength, and centrifugation time and rate in DLLME step were studied. All experiments were performed triplicate.

*Optimization of HLLE step*

*Type of extraction/disperser solvent*

In this study, an extractant has a double role: (i) as an extractant in HLLE step, and (ii) as a disperser in the next preconcentration step (DLLME). This solvent is selected on the basis of its miscibility with the organic phase (extraction solvent of DLLME) and aqueous phase (to form a homogenous solution), its ability to produce a two–phase system upon dissolution of a salt, and its high extraction efficiency for the selected drugs from the aqueous solution. According to these criteria, ACN, acetone, *iso*-propyl alcohol, and DMF (2 mL of each solvent) were tested as the extraction solvents in HLLE. The results indicated that only ACN formed a two-phase system, while other solvents could not be separated from the aqueous solution by passing the homogenous solution through the tube filled with sodium chloride. Hence, ACN was selected as extraction/disperser solvent in the subsequent experiments.

*Volume of extraction/disperser solvent*

**Figure 1:**
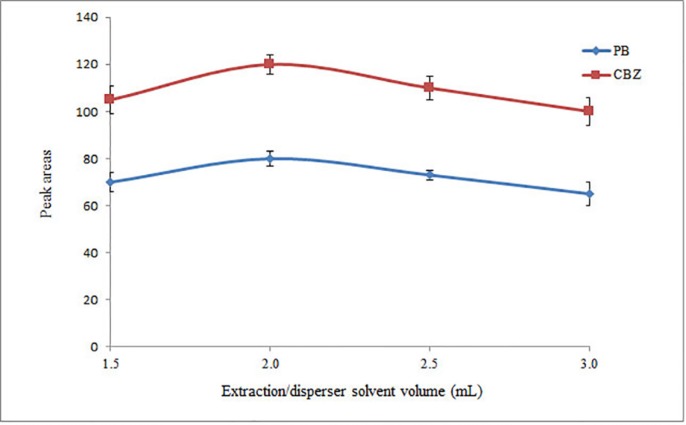
Selection of extraction/disperser solvent volume. Conditions: Extraction/disperser solvent, ACN; sample volume, 5 mL diluted drug free urine sample spiked with the analytes at a concentration of 1 mg L–1 of each analyte; pH, 5; flow rate, 0.5 mL min-1; extraction solvent, chloroform (40 µL); and centrifuge rate and time, 6000 rpm and 6 min, respectively. The error bars indicate the minimum and maximum of three determinations

Extraction/disperser solvent volume is another factor that affects extraction efficiency. To evaluate the effect of extractant volume, 1.5, 2.0, 2.5, and 3.0 mL of ACN were applied for extraction of the target analytes. The volumes of the collected ACN in HLLE step were 0.60, 1.0, 1.4, and 1.8 mL for the mentioned volumes, respectively. As can be seen in Figure 1, the peak areas of the analytes increase by increasing the volume of ACN to 2.0 mL and then decrease with further increases in volumes. It is noted that in all cases, 1.0 mL of the collected organic phase was used in the second step (DLLME step). However, in the case of 1.5 mL ACN, only 0.60 mL ACN was collected after passing the whole of the solution through the tube. In that case, it was mixed with 0.4 mL pure ACN before performing the following DLLME step. Finally, 2.0 mL ACN was selected as the optimum volume of extraction/disperser solvent for the following experiments.

*Study of pH*

**Figure 2:**
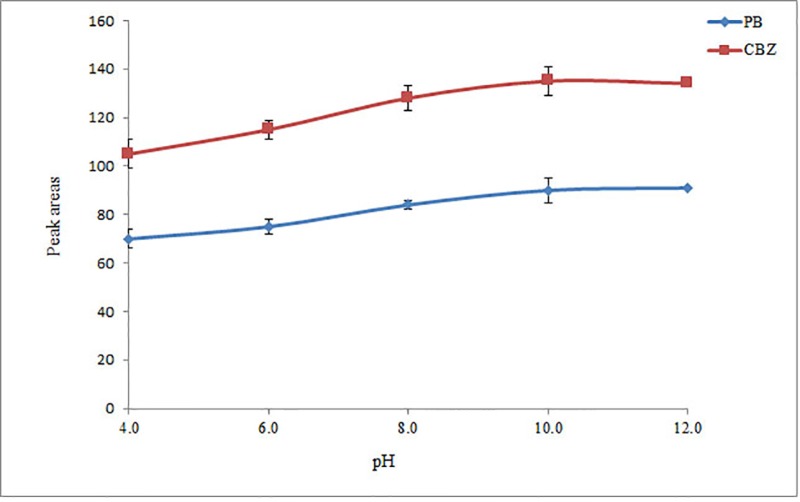
Study of pH. Conditions: The same as used in Figure 1, except 2 mL ACN was used as the extraction/disperser solvent volume

PB and CBZ are basic compounds that undergo protonation in an acidic medium. Consequently, their extraction into an organic solvent is favored in a basic medium, being independent on the used extraction technique. The pH of the aqueous phase was adjusted in the range of 4 to 12 (in 2-unit intervals) with 0.1 M HCl or NaOH. It is observed (Figure 2) that extraction efficiency of the selected drugs in the acidic solutions is low. Based on the obtained results, extraction of the analytes at the pH range of 10-12 is favorable. Hence, 10 was selected as the optimum pH.

*Flow rate study*

**Figure 3:**
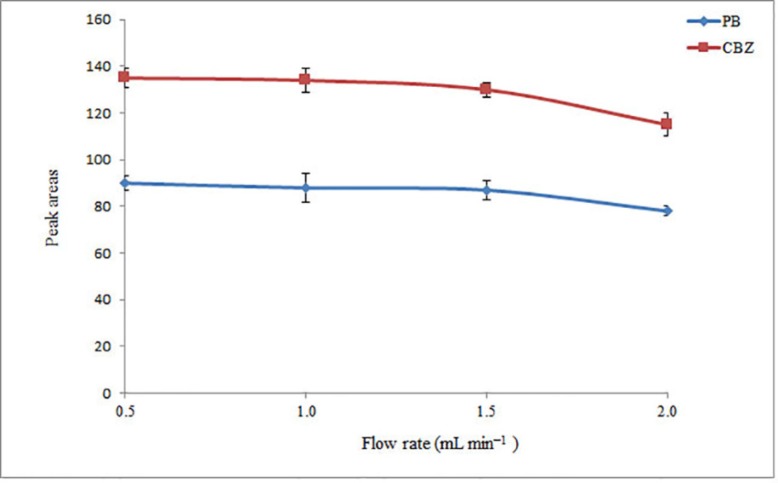
Effect of flow rate on the extraction efficiency of the method. Conditions: The same as used in Figure 2, except pH of sample was adjusted in 10

The sample flow rate through the tube filled with NaCl is one of the most important parameters allowing ER of the presented method to be improved. Indeed, by reducing the flow rate, more salt is dissolved in the aqueous phase and phase separation is facilitated. However, at low flow rates the analysis time will be relatively long. Therefore, the influence of sample flow rate on the analytical signals of the analytes was investigated in the range of 0.50–2.0 mL min^−1^ in 0.5 mL intervals. The obtained results (Figure 3) show that the analytical signals are nearly constant up to 1.5 mL min^–1^ and then decrease at 2.0 mL min^-1^. This indicates that an inadequate extraction of the analytes from the aqueous phase is obtained at flow rates higher than 1.5 mL min^–1^. This behavior can be explained because the amount of the dissolved salt in the aqueous phase is decreased at higher flow rates and leads to a decrease in extraction efficiency. Therefore, a flow rate of 1.5 mL min^−1^ was chosen for the further analysis.

*Optimization of DLLME*

*Selection of extraction solvent*

**Figure 4:**
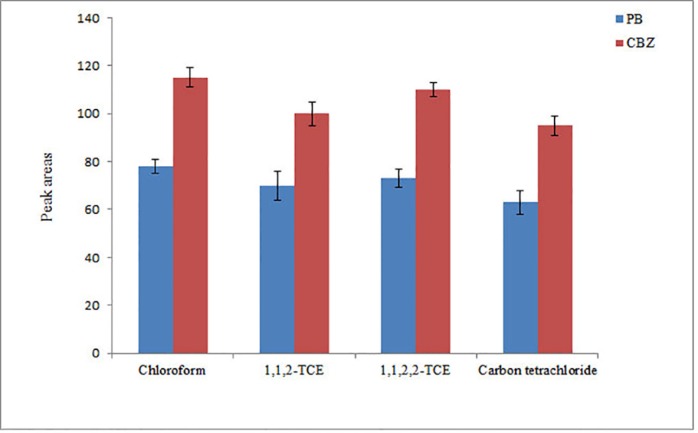
Study of extraction solvent type in DLLME. Conditions: The same as used in Figure 3, except flow rate was 1.5 mL min-1

The extraction solvent in this step is important in the optimization of the proposed method. For the selection of this solvent, its low solubility in water, high affinity to analytes, and higher density than water are important. In this work, chloroform, 1,1,2-TCE, 1,1,2,2-TCE, and carbon tetrachloride were investigated as the extraction solvents. It is noted that to achieve a same volume of the settled phase (10 ± 0.5 µL), 40, 38, 35, and 42 µL of each solvent was used, respectively. From the results in Figure 4, it is found that the analytical signals of the analytes obtained using chloroform are higher than the other tested extraction solvents. Thus, chloroform was selected as an extraction solvent for the further experiments.

*Extraction solvent volume*

Generally, in a DLLME procedure, the volume of the extraction solvent is an effective parameter on the extraction efficiency. At high volumes of the extraction solvent, generally the extraction recovery of the DLLME procedure is increased. On the other hand, at those volumes, the extracted analytes are diluted and therefore, the EFs is decreased. In this study, 30, 40, 50, and 60 µL of chloroform were evaluated for the DLLME step. The obtained results showed that the analytical signals were increased when the chloroform volume increased up to 40 µL and then decreased with further increase of chloroform volume, due to decrease of EFs in the DLLME step. Therefore, 40 µL of chloroform was selected as the optimized extraction solvent volume.

**Figure 5:**
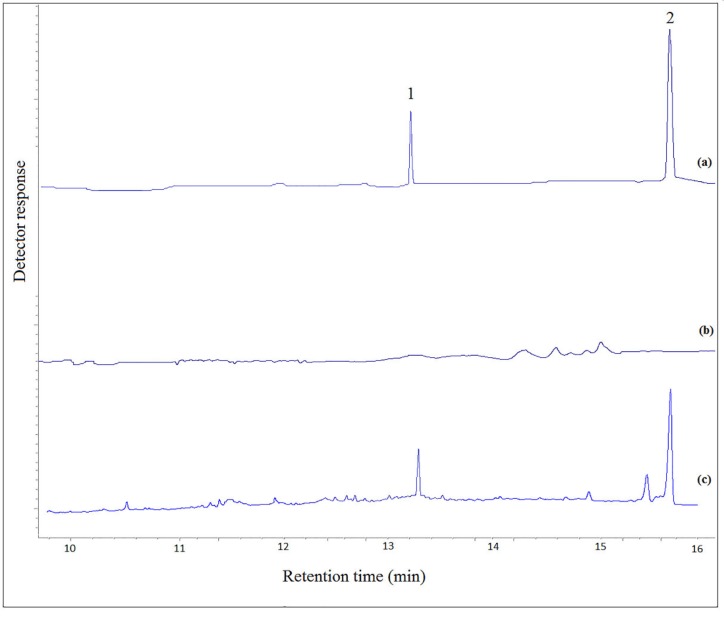
Typical GC–FID chromatograms of: (a) a standard solution prepared in chloroform (100 mg L−1, each analyte), (b) drugs free urine, and (c) drugs free urine spiked with the analytes at a concentration of 1 mg L-1 of each analyte. In chromatogram (a) direct injection was used. In other cases the proposed method was performed on them and 1 µL of the final organic phase was injected into the separation system

**Table 1:** Quantitative features of the proposed method for the selected drugs

**Analyte**	**LOD** a	**LOQ** b	**LR** c	**r** **2 ** d	**RSD (%)** e		**ER ± SD** f	**EF ± SD** g
					**Intra–day**	**Inter– day**		
PB	0.017	0.056	0.06 – 100	0.999	7	8	45 ± 1	100 ± 2
CBZ	0.010	0.033	0.04 – 100	0.998	5	5	54 ± 2	135 ± 5

a Limit of detection (S/N = 3**) (**µg mL–1).

c Linear range (µg mL–1).

d Coefficient of determination.

e Relative standard deviation (n = 6, C = 1 µg mL–1 of each drug) for intra–day and (n = 4, C = 1 µg mL–1 of each drug) for inter–day precisions.

f Extraction recovery ± standard deviation (n = 3).

g Enrichment factor ± standard deviation (n = 3).

**Table 2:** Comparison of the presented method with other methods used in preconcentration and determination of the studied drugs

**Drug**	**Sample**	**RSD (%)** a	**LR** b **(µg L-1)**	**LOD** c	**LOQ** d	**Method**	**Ref.**
BP	Urine	≤ 10.76	-	44	80	LLE-LC-MS/MS^e^	(24)
							
CBZ	Urine	6.1	5-200	0.0015	0.005	SA-DLLME-HPLC-UV^f^	(25)
							
BP	Urine	7.6	0.08-40	-	0.08	SBSE-HPLC-UV^g^	(26)
CBZ		8.8	0.08-40	-	0.08		
PB	Urine	7	0.06-100	0.017	0.056	Proposed method	
CBZ		5	0.04-100	0.010	0.033		

a Relative standard deviation.

b Linear range (µg mL^-1^).

c Limit of detection (µg mL^-1^).

d Limit of quantification (µg mL^-1^).

e Liquid–liquid extraction- liquid chromatography tandem mass spectrometry.

f Surfactant assisted dispersive liquid–liquid microextraction- high performance liquid chromatography- UV detection.

g Stir bar sorptive extraction-high-performance liquid chromatography-UV detection.

**Table 3:** Relative recoveries of PB and CBZ obtained by the proposed method in urine samples spiked at four concentrations

**Analyte**	**Nominal concentration (µg mL** **-1** **)**	**Found concentration (µg mL** **-1** **) ± standard deviation (n = 3)**	**Relative recovery**
PB	1	1.062 ± 0.051	106 ± 5
CBZ		0.984 ± 0.024	98 ± 2
PB	5	4.810 ± 0.152	96 ± 3
CBZ		4.840 ± 0.136	97 ± 3
PB	15	14.641 ± 0.401	98 ± 3
CBZ		15.514 ± 0.355	103 ± 2
PB	20	20.642 ± 0.842	103 ± 3
CBZ		19.551 ± 0.725	100 ± 2

*Influence of salt addition*

Ionic strength was examined by addition of sodium chloride. To evaluate the effect of ionic strength, various concentrations of sodium chloride, from 0 to 12%, w/v, were studied. The obtained results showed that the extraction efficiency slightly increased when the salt concentration was increased to 10% w/v, but it decreased as the salt concentration was further increased to more than 10% w/v. This phenomenon can be explained by the fact that the addition of a small amount of salt can enhance the partition of analytes in the extraction solvent, thereby improving the extraction recovery. However, high salt concentration can increase the viscosity of the aqueous phase, thus slowing the decreased mass transfer of the analytes and the ER. For these reasons, 10% w/v, NaCl was chosen for the subsequent studies.

*Centrifugation time and rate*

Centrifugation time and rate in DLLME step were studied in the ranges of 2–6 min and 2000–8000 rpm, respectively. The results indicated that centrifugation time and rate had no effect on the extraction efficiency because after mixing solvents (aqueous phase, extraction solvent, and dispersive solvent) the equilibrium status was achieved in a few seconds due to large contact area between tiny droplets of the extraction solvent and sample. Thus, centrifugation was only used to help the cloudy solution to be broken and the extraction solvent to settle at the bottom of the tube. Therefore, centrifugation time and rate of 6 min and 6000 rpm, respectively, were chosen to ensure that the transfer of droplets to the bottom of the tube is complete

*Analytical performance of the proposed method*

Under the optimized conditions, the analytical performance of the proposed method was evaluated and the linear range (LR), relative standard deviation (RSD), limit of detection (LOD), limit of quantification (LOQ), ER, and EF are listed in Table 1. As can be seen in Table 1, the linearity is excellent considering the wide range studied, and the r^2^ is ≥0.998 for the selected drugs. 

The LODs and LOQs were estimated by extraction of the selected drugs from the spiked drug free urine with low concentration levels and injecting the obtained final organic phase into the instrument to give a signal to noise ratio of 3 or 10, respectively. The LODs for CBZ and PB were 0.010 and 0.017 µg mL^-1^ and the LOQs were 0.033 and 0.056 µg mL^-1^, respectively. The RSDs% for intra- and inter-day precision at a concentration of 1 of each drug were ≤8% indicating the good precision achieved by the proposed procedure. The ERs% were calculated based on Equation 1 were 45 and 54% for PB and CBZ, respectively. The corresponding values for EFs (Equation 2) were 100 and 135%.

*Comparison of the proposed method with others*

The quality factors (RSD, LOD, LOQ_, _and LR) of the proposed method for the analysis of BP and CBZ drugs are compared with those of other previously reported procedures for the determination of the same drugs in Table 2. The results indicate that the proposed procedure has good repeatability than other methods. The LOD_S_ and LOQ_S_ obtained for the analytes by the presented procedure are lower than those of other methods, except the second method. Also, the EF_S_ obtained by the proposed method (100 and 135 for PB and CBZ, respectively) are good considering the low sample volume (2.5 mL) used in this study. These results indicate that the proposed method is repeatable and sensitive and it can be utilized for the extraction, preconcentration, and determination of PB and CBZ in urine.

*Application to urine samples*

The performance of the new analytical method was tested in different urine samples spiked with the selected analytes. The urine samples were extracted by the proposed method and then analyzed using GC-FID. The results of the quantitative analysis of the urine samples are listed in Table 3. The typical GC-FID chromatograms of a standard solution of the selected drugs in chloroform, drug free urine sample (blank urine), and urine sample spiked with the analytes are shown in Figure 5. There is no interfering peak in the retention times of the analytes in blank urine.

## Conclusion

A simple method by combining HLLE performed in a narrow tube and DLLME followed by GC-FID has been developed for the analysis of two antiepileptic drugs in urine samples. Under the established conditions, the presented method showed good linearity, acceptable repeatability, and low LODs. The proposed method provided many advantages such as simplicity, rapidity, low cost, high sensitivity, and high EFs for the determination of the target drugs at trace levels in complex matrices.
